# Cleaning and disinfection of transport crates for poultry – comparison of four treatments at slaughter plant

**DOI:** 10.1016/j.psj.2021.101521

**Published:** 2021-10-10

**Authors:** T. Dzieciolowski, S. Boqvist, J. Rydén, I. Hansson

**Affiliations:** ⁎ Department of Biomedical Sciences and Veterinary Public Health, Swedish University of Agricultural Sciences, Uppsala SE-750 07, Sweden; † Department of Energy and Technology, Swedish University of Agricultural Sciences, Uppsala SE-750 07, Sweden

**Keywords:** *Campylobacter*, transport crate, poultry, cleaning, disinfection

## Abstract

Transport crates for poultry can contribute to the spread of pathogens, with those of public health interest, for example, *Campylobacter*, being of particular importance. A strict cleaning procedure and use of an effective disinfection method for transport equipment are thus important to avoid introduction of *Campylobacter* to chicken and poultry farms, particularly during flock thinning. This study evaluated the efficacy of the disinfection procedure currently in use at one of the largest slaughter plants in Sweden and compared the effects with those of other disinfection methods. The evaluation was based on treatment ability to reduce the presence and amount of indicator bacteria belonging to the family *Enterobacteriaceae* and total aerobic bacteria. In 4 trials, sodium hypochlorite, peracetic acid, and drying with hot air, with or without sodium hypochlorite for final disinfection, were compared. The analysis was based on 40 cotton swab samples taken in each treatment, 20 after the soaking stage and 20 after the final disinfection step.

The results showed that use of a chemical disinfectant in combination with drying with hot air (dehumidifier) was the most effective treatment, with an average reduction of 3.4 log for total aerobic bacteria and 3.8 log for *Enterobacteriaceae.* Since all crates treated with hot air were dry, transport conditions for the birds also improved, particularly in cold weather. A disadvantage is that this treatment is energy-consuming and would require substantial technical changes to the current cleaning process, increasing operating costs at the slaughter plant. However, considering the contribution of improved crate cleaning to overall hygiene control within the poultry supply chain and the beneficial effect on animal welfare, the costs may be justified.

## INTRODUCTION

Campylobacteriosis is the most frequently reported zoonosis within the European Union, with more than 200,000 confirmed cases in 2019, representing more than 50% of all reported human cases of zoonotic infections. Chicken and chicken products are known to be the major sources of *Campylobacter* infection for humans (Authority et al., 2021). Poultry meat can become contaminated with *Campylobacter* during slaughter if the live chickens are intestinal carriers of the organism. There are indications that the practice of thinning, when a part of a chicken flock is slaughtered while the remaining chickens are left to grow for an additional period, is a potential source of *Campylobacter* spread (Hansson et al., 2010). Transport crates are often contaminated with faeces and mostly reused on the same day for transportation of birds from different farms (Carr et al., 1999; Wilkins et al., 2003). This makes them potential vehicles for transmission of *Campylobacter* between broiler flocks in conjunction with thinning (Slader et al., 2002; Hansson et al., 2005). There is also evidence that current commercial washing systems for crates do not reduce microbial contaminants such as *Campylobacter* (Slader et al., 2002; Atterbury et al., 2020). Hence there are risks of bacterial spread (Allen et al., 2008b; Frosth et al., 2020; Hertogs et al., 2021). A thorough cleaning procedure and use of an effective disinfection method for transport equipment are thus important in preventing introduction of *Campylobacter* to chicken groups during thinning. In addition, areas with a colder climate, such as the Nordic countries, require the transportation equipment to be dry, since wet crates at low temperatures have a negative impact on animal welfare due to the increased risk of hypothermia. A solution that ensures both clean and dry crates would therefore be optimal from a biosecurity and welfare perspective.

Several studies have shown that pathogenic bacteria, such as *Campylobacter* spp. and *Salmonella* spp., remain present on transport crates for chickens even after cleaning and disinfection (Hansson et al., 2005; Atterbury et al., 2020). However, in applied research indicator bacteria are often used instead of pathogenic bacteria. *Enterobacteriaceae* in general or *E. coli* in particular has been proven to positively correlate when compared to the concentration of *Campylobacter* (Williams and Ebel, 2014; Parette, 2018; Roccato et al., 2018).

*Enterobacteriaceae* and total aerobic bacteria counts has been therefore used as indicators of fecal contamination, to evaluate efficacy of disinfection methods on transport crates and to estimate the risk of presence of pathogenic bacteria (Allen et al., 2008a; Atterbury et al., 2020).

Many studies have investigated the effect of cleaning methods on transport crates. For example, a study in the United Kingdom tested different cleaning and disinfection procedures for crates in an experimental rig, obtaining useful information about potential strategies that can be implemented in practice (Allen et al., 2008a). Following that research, a study at commercial scale was carried out, resulting in reduced *Enterobacteriaceae* and *Campylobacter* (Atterbury et al., 2020). Other studies have shown that the bacterial load can be reduced by ultraviolet LED light (Moazzami et al., 2021) and that using forced hot air to dry chicken transport crates results in low numbers of *Campylobacter, Escherichia coli* and coliforms (Berrang et al., 2011).

For chemical disinfection of transport crates, there are different active substances that can be applied. Two commonly used disinfectants within the food industry are sodium hypochlorite and peracetic acid, which are inexpensive, relatively nontoxic and have a broad spectrum of antimicrobial activity (Fukuzaki, 2006; Gawande et al., 2013).

This study was performed at one of the largest poultry slaughter plants in Sweden. The aims were to evaluate how efficiently the plant's current disinfection procedure for transportation crates reduces levels of the *Enterobacteriaceae* and total aerobic counts, and to compare the effects with those of other disinfection methods.

## MATERIALS AND METHODS

### Description of Transport Crates and Their Usage

**Figure 1:**
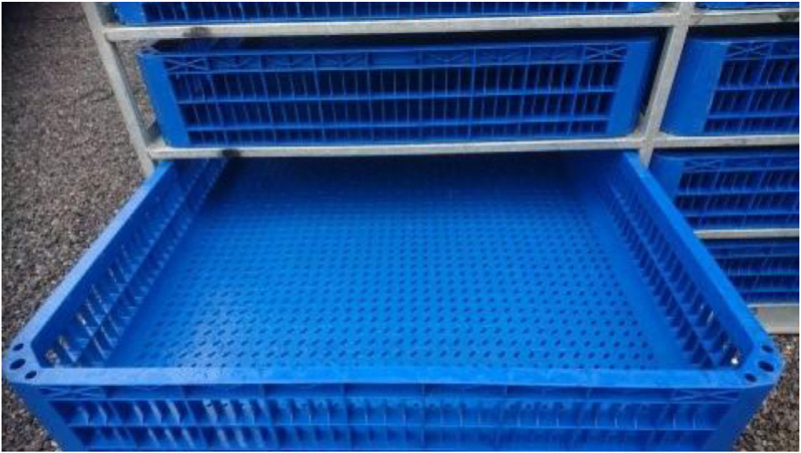
High-density polyethylene crates in a steel frame used to transport chickens to slaughter.

This study was performed at a Swedish poultry processing plant with a slaughter capacity of 12,000 birds per hour. The transport crates (Linco Food Systems, Trige, Denmark) used at the plant are composed of high-density polyethylene, with approximate dimensions 110 mm × 1163 mm × 1163 × 240 mm and fitted as drawers in steel frame (Figure 1). The crates have many small perforations in the base for drainage and larger perforations in the sides to facilitate air movement during transportation. A module design facilitates loading of live birds on farms to transport them to the slaughter plant. Each module, made of steel frame, contains 10 crates stacked in 2 adjacent stacks, with 5 crates per stack. A forklift is used for unloading the modules at the slaughter plant. The modules are then transported to a stunning unit by conveyor belt. At the stunner, the crates are removed, and the steel modules are transported on to a cleaning area, while the crates containing the birds enter the stunner. After the stunning procedure, the crates of stunned birds are turned upside-down and the birds are placed on a conveyor belt that transports them to the hanging area, while the empty crates are transported to a cleaning area.

### Cleaning of Transport Crates

The cleaning system (Linco Food Systems) used for transport crates at the slaughter plant consists of the following 5 fully automated steps:

■ Prewashing is done using a set of spray nozzles, which apply cold water from above and below each crate.
■ To soften attached debris, the transport crates are then soaked for 90 s in an elongated tank filled with cold water with 0.5% (v/v) sodium hypochlorite (Cip Alka CL, Novodan, Kolding, Denmark) added as a detergent.
■ The main washing takes place in 3 consecutive washing modules using high-pressure nozzles spraying cold water on all surfaces of the crate.
■ The crates are dried and surplus water is removed by cold air jets applied in a drying module equipped with 5 large side-channel blowers, each 8.5 kW. Using high pressure and specially designed air blades, the crates are hit with air jets at a speed of approximately 235 km/hour.
■ Finally, the crates are disinfected with sodium hypochlorite (0.5% v/v) by a set of spray nozzles, which apply the solution from above and below to each passing transport crate for approximately 1 s.

### Study Design

A total of 4 different trials (A–D) were carried out in the slaughter plant. Two of the trials (A and B) tested 2 chemical disinfectants commonly used by the food industry. In trial A (reference treatment), sodium hypochlorite (Hypochlor Des, Novodan) at 0.5% (v/v) was used, representing current practice at the slaughter plant. Trial B included the same automated cleaning step as trial A, but in the last step the crates were disinfected with 0.5% (v/v) peracetic acid (Oxidan Extra, Novadan, Kolding, Denmark).

The other 2 trials (C and D) tested alternative treatments based on the principle of drying the crates with hot air. For this purpose, a test rig was designed and built adjacent to the slaughter plant. A dehumidifier (model ML17, Munters, Kista, Sweden) was installed on a steel container (2,400 mm in width × 2900 mm in length × 2500 mm high). One transportation module with 10 crates, cleaned according to the usual routine, was placed in the container. The crates were dried for 2 h, the maximum time available between cleaning and reloading transport crates on transportation trucks to maintain an even flow in the loading process. In trial C, the channel blowers were disconnected, and the transport crates were dried with hot air without using any chemical disinfectant in the last step of the cleaning process. In trial D, the combined effects of hot air and disinfectant were studied. The drying procedure in trial D was the same as for trial C and the disinfectant used was the same as for trial A, that is, sodium hypochlorite at 0.5% (v/v). Steps of the cleaning process that crates went through during each trial are shown in Table 1.

**Table 1:** Steps of the cleaning process that crates went through during each trial, in the order they took place.

Step trial	Prewashing	Soaking	Main wash	Surplus water removal with channel blowers	Disinfection			Drying with dehumidifier
					Sodium hypochlorite	Peracetic acid	None	
A	✓	✓	✓	✓	✓			
B	✓	✓	✓	✓		✓		
C	✓	✓	✓				✓	✓
D	✓	✓	✓		✓			✓

### Sample Collection

During each trial, the inner base of the crates was sampled with a sterile cotton swab measuring 10 cm × 10 cm (Wellkang Ltd, London, UK), using a gloved hand. The swabs were moistened immediately before sampling, with 60 mL buffered peptone water (**BPW**) (Oxoid CM0509; Basingstoke, UK). One swab was used to sample the whole inside base by wiping the crate vertically with one side of the swab and horizontally with the other side.

The selected crates were marked with tape and sampled at 2 stages during the washing process, immediately after the soaking stage (referred to as ‘presample’) and after the final disinfection stage (referred to as ‘postsample’) for trial A and B, or the drying stage for trial C and D. Each cotton swab was then placed in a sterile plastic bag and 60 mL BPW were added. Each trial was carried out during 2 consecutive days. Ten pre- and 10 postsamples were taken during each day, on 3 different occasions on during each sampling day that is 20 pre- and 20 postsamples for each trial giving 80 pre- and 80 postsamples in total.

All samples were transported in plastic insulated cooler boxes with frozen gel packs. The temperature was checked upon arrival and analyzed within 24 h. Samples with a minimum temperature of 0°C (not frozen) and at a maximum temperature of 4°C were accepted for analyses.

### Analysis of Total Number Aerobic Bacteria

The samples were analyzed for total number of live, aerobic bacteria according to NMKL-method 86 (5th Ed., 2013). In brief, 90 mL BPW were added to the swab sample and stomached (easyMIX Lab Blender, AES-Chemunex, Weber Scientific, Hamilton, NJ) for 1 min. A 10-fold serial dilution in 0.1% (v/v) peptone water (Oxoid) was prepared and 1.0 mL from each dilution was mixed carefully with 10 to 15 mL of plate count agar (**PCA**) (Oxoid in a Petri dish (9 cm diameter). After agar solidification, the plates were incubated at 30.0°C for 72 ± 7 h. Plates with 25 to 250 colonies were selected for quantification, since these are considered to give the most accurate microbiological results.

### Analysis of Enterobacteriaceae

Analysis for bacteria belonging to the family *Enterobacteriaceae* was performed according to NMKL 144 (3rd Ed., 2005). The previously prepared 10-fold dilutions were also used to estimate counts of *Enterobacteriaceae* in samples. From each dilution, 1.0 mL was mixed carefully with 10 to 15 mL violet red bile glucose agar (**VRBG**) (Becton, Dickinson and Company, Sparks, MD) in a Petri dish and left to solidify, and then an overlay of 5 mL VRBG was added and the plates were incubated at 37 ± 1°C for 24 ± 2 h. The numbers of suspected bacteria belonging to the *Enterobacteriaceae* were counted on plates with 15 to 150 colonies. Five colonies preliminarily identified as *Enterobacteriaceae* were cultured on blood agar and incubated at 37 ± 1°C for 24 ± 2 h. Presence of bacteria belonging to the *Enterobacteriaceae* was confirmed by oxidase test and the number of *Enterobacteriaceae* was expressed as log colony-forming units (**CFU**) per mL. The detection limit was log 1.0 CFU/mL.

### Statistical Analyses

The data obtained in the study were compiled and analysed using Excel and R Core Team 2021. Bacterial counts (CFU/mL) were log_10_-transformed. Standard deviations of bacterial reductions for each treatment were calculated. For the analysis, a linear mixed model was employed, and multiple comparisons were made by Tukey's HSD.

## RESULTS

### Total Aerobic Bacteria

**Figure 2:**
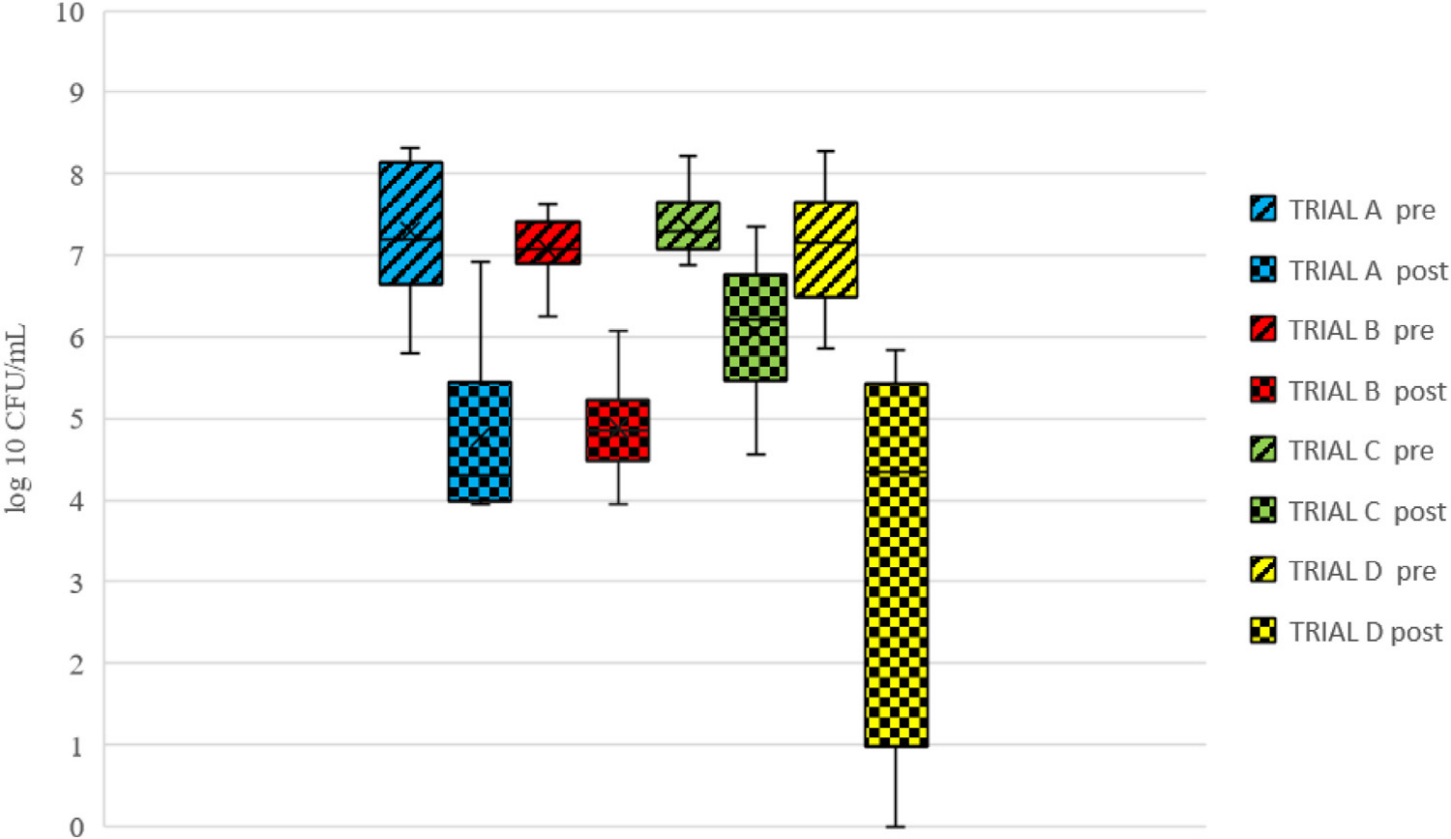
Mean counts of total aerobic bacteria (log CFU/mL) detected on swab samples taken from chicken transport crates before (pre-) and after (post-) cleaning in trials A–D.

Before crate cleaning and disinfection, the total number of aerobic bacteria varied from 5.8 to 8.3 log CFU/mL, with a mean of 7.3, 7.1, 7.3, and 7.0 log CFU/mL in trial A, B, C and D, respectively (Figure 2). One sample from trial C was excluded due to mix-up of the presample and postsample during processing. No significant difference was observed between the different trials regarding the number of aerobic bacteria on the crates before the different disinfection trials.

The greatest mean reduction in total aerobic bacteria was 3.4 log and was achieved in trial D (Table 2). A significant difference was observed for total aerobic bacteria between trials D and C (*P* < 0.001) and trials D and B (*P* = 0.01), but not between trials A and D (*P* = 0.09; Figure 2). A reduction in total aerobic bacteria was observed in the samples from all individual crates. There was in addition a significant effect due to sampling day (*P* < 0.001).

**Table 2:** Log reduction (mean and standard deviation [SD]) in total aerobic bacteria counts in swab samples taken from chicken transport crates before and after the cleaning process in trials A–D.

Trial	Description of trial	Total aerobic bacteria	
		Reduction	SD
A	Sodium hypochlorite 0.5% (v/v)	2.2	1.1
B	Peracetic acid 0.5% (v/v)	2.2	0.8
C	Dehumidifier 2 h, without disinfectant	1.5	0.8
D	Dehumidifier 2 h, sodium hypochlorite 0.5% (v/v)	3.4	2.4

### Enterobacteriaceae

**Figure 3:**
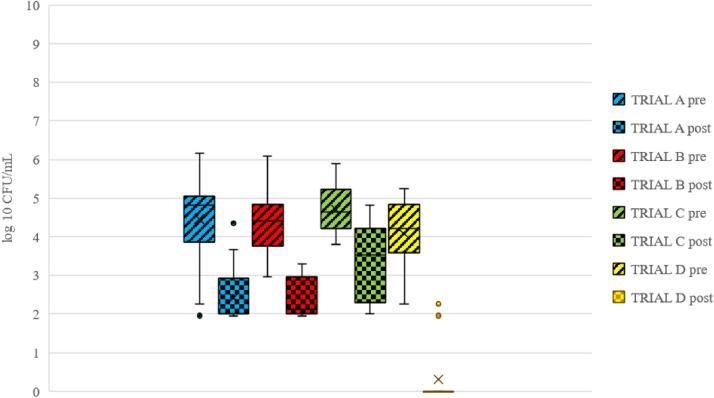
Mean counts of *Enterobacteriaceae* (log CFU/mL) detected on swab samples taken from chicken transport crates before (pre-) and after (post-) cleaning in trials A–D.

Before crate cleaning and disinfection, the total number of *Enterobacteriaceae* varied from 2.0 to 6.2 log CFU/mL, with a mean of 4.5, 4.3, 4.8, and 4.1 log CFU/mL in trials A, B, C and D, respectively (Figure 3). Three samples from trial C were excluded due to mix-up of presample and postsample during processing. The greatest mean reduction in *Enterobacteriaceae*, 3.8 log, was achieved in trial D (Table 3). There was a significant difference (*P* < 0.001) in the average reduction seen for *Enterobacteriaceae* between trial D and all the other trials (Table 3). In fact, in 85% (17 of 20) of postsamples in trial D, *Enterobacteriaceae* were not detectable. In the three samples in which bacteria colonies were found, the levels were similar to those in the other trials (Figure 3). There was in addition a significant effect due to sampling day (*P* < 0.05).

**Table 3:** Log reduction (mean and standard deviation [SD]) in *Enterobacteriaceae* counts in swab samples taken from chicken transport crates before and after the cleaning process in trials A–D.

Trial	Description of trial	*Enterobacteriaceae*	
		Reduction	SD
A	Sodium hypochlorite 0.5% (v/)	1.6	1.3
B	Peracetic acid 0.5 % (v/v)	1.6	1.0
C	Dehumidifier 2 h, without disinfectant	1.7	1.5
D	Dehumidifier 2 h, sodium hypochlorite 0.5% (v/v)	3.8	1.2

## DISCUSSION

The greatest reduction in bacteria on chicken transport crates in this study was achieved by applying hot forced air (dehumidifier) combined with a commonly used disinfectant (sodium hypochlorite), which gave an average reduction of 3.4 log for total aerobic bacteria and 3.8 log for *Enterobacteriaceae.* The procedure of drying the crates alone without subsequent chemical disinfection (trial C) gave an average reduction of 1.5 log for total aerobic bacteria and 1.7 log for *Enterobacteriaceae*. Since no significant difference in *Enterobacteriaceae* was found between trial C and trials A and B, it appears that all these treatments were equally effective (or ineffective) in this instance. The significant difference in reduction between sampling days is hard to explain since the sampling procedure, the time of the delivery to the lab as well as the analyses were the similar for both sampling occasions. That might be an indication for either the cleaning system not operating consistently at all times, or that the outcome of cleaning procedures depends on the contamination level of the crates. Transport duration varied from half an hour up to 3.5 h. The longer transport duration the drier feces, which might be harder to remove from crates´ surface (personal observations).

There is no established standard for required level of cleanliness for transport crates, but it has been suggested that a 2 log reduction in microbial contamination is desirable and a 4 to 5 log reduction is clearly satisfactory (Allen et al., 2008a). In our study, the reduction in microbial contamination did not reach that satisfactory target in any of the trials. According to Swedish Standard SS-EN 14349:2007 for chemical disinfectants used in veterinary applications, a reduction of 5 log in different test bacteria should be achieved. However, that value applies to smooth surfaces such as examination tables and other nonporous working surfaces, and thus does not seem to be a suitable guide for chicken transport crates. Our results support findings in other studies that achieving a fully satisfactory reduction in bacterial load in real commercial operations is not an easy task (Atterbury et al., 2020).

Experimental simulations suggest that drying transport crates for an extended period could be effective in reducing the microbial load (Berrang and Northcutt, 2005). *Campylobacter* is sensitive to drying (Doyle and Roman, 1982), which would make such treatment useful against one of the most important pathogens for the poultry industry. Furthermore, there are indications that using forced hot air to dry the crates could facilitate treatment in practice to such an extent that it can become commercially applicable. For instance, flowing air at approximately 50°C for 15 min has been successful in reducing the numbers of *Campylobacter* to an undetectable level (Berrang et al., 2011). However, these findings have been made under laboratory conditions and, to our knowledge, not tested under ordinary commercial conditions. In a study which attempted to dry transport crates in conditions mimicking real commercial conditions using a test rig with air jets (Allen et al., 2008a), an unsatisfactorily low reduction in microbial contamination was obtained, probably due to short treatment time (60 s), although in that case the air used was not heated. In the present study, undertaken in a real high-throughput commercial operation, hot air in combination with a disinfectant (trial D) was effective in reducing the bacterial load. The usage of heated air as disinfection means on a big-scale requires a huge air movement within drying area. Furthermore, the heated air recirculates in a closed loop thereby lowering the operating costs. That may pose a risk for recontamination of the drying area which in turn could potentially lead to contamination of crates previously free from the pathogen. This might be the case when the cleaning processes in previous stages of are insufficient and highly contaminated crates are placed in the drying area. The risk could be mitigated by installing filters for returning air.

Other approaches, such as applying hot water in combination with detergent and disinfectant, tested in an experimental rig (Allen et al., 2008a) and followed up in a study on commercial scale (Atterbury et al., 2020), have given promising results. For instance, a combination of soaking in water at 55°C, brushing and washing at 60°C, followed by use of a disinfectant resulted in at least a 4 log reduction in the number of *Enterobacteriaceae*, but was less effective in reducing the total number of aerobic bacteria (Allen et al., 2008a). In the follow-up study on commercial scale, use of heated water (60°C) in combination with a detergent rinse and disinfectant spray resulted in a 3.6 log reduction in *Enterobacteriaceae* and a 3.8 log reduction in *Campylobacter* (Atterbury et al., 2020), which is in line with our findings. Since both that study and ours were performed in a real commercial operation, combined heat treatment and detergent use may be a feasible solution in practice. However, solutions using hot water instead of air lack the benefit of improved animal welfare from dry crates.

In a study by Moazzami et al. (2021), applying ultraviolet LED light to chicken transport crates under laboratory conditions gave a 2.0 log reduction in *Campylobacter jejuni*, a 1.5 log reduction in *Enterobacteriaceae* and a 1.4 log reduction in total aerobic bacteria when the irradiation time was 1 minute. Extending the irradiation time to 3 min resulted in a 3.1 log, 1.8 log and 1.6 log reduction, respectively (Moazzami et al., 2021). However, practical implementation of ultraviolet LED light technology might be a problem due to difficulties in achieving exposure of the whole surface of the crates to LED light, given the complicated construction of transport crates.

Choosing the optimal disinfectant for transport crates is another issue. Sodium hypochlorite is commonly used because of its broad antibacterial spectrum, rapid bactericidal action, ease of use, stability in solution, relative nontoxicity to humans, low cost, and acceptable cleaning action (Fukuzaki, 2006). Another useful property of sodium hypochlorite is its ability to act as a detergent, which can be exploited by adding it to the soaking tank in the second stage of the cleaning process to reduce the organic load before the next stage. However, using sodium hypochlorite as the disinfectant in the final stage of the process may not be the optimal solution in an environment where organic matter is present. Water used in the cleaning system currently in use at the slaughter plant where all our trials took place is connected in a counter-flow arrangement, where water added in the last washing module flows back to the soaking tank, while water used in the first and second washing modules is recirculated. The water is filtered before being reused, but some organic debris may still be present. This study showed that peracetic acid can be used instead of sodium hypochlorite because of its similar antibacterial and practical properties and because its efficacy is not impaired in the presence of organics. We observed similar reductions in total aerobic bacteria and *Enterobacteriaceae* for both sodium hypochlorite and peracetic acid (trials A and B), leading us to conclude that they are equally effective, although the microbial reduction is not as high as desired in either case.

Our results suggest that a system involving use of a chemical disinfectant in combination with drying with hot air has good potential for reducing the microbial load on transport crates for chickens and can thus mitigate the risk of *Campylobacter* being introduced into chicken flocks during thinning. Furthermore, all transport crates subjected to hot air treatment (using a dehumidifier; trials C and D) were fully dry, thus providing better transport conditions for the birds in cold weather conditions. However, using hot air on a large scale in practical conditions is energy-consuming and implementation would require substantial technical changes in the current cleaning process, inevitably increasing operating costs for the slaughter plant. Nevertheless, considering the contribution of improved crate cleaning to overall hygiene control within the poultry supply chain in general, especially with regard to *Campylobacter*, and the beneficial effect on animal welfare, the costs may be justified.
